# Trends in Prevalence and Predictors of Anemia in Adolescents Between the Ages of 15 and 19 Years in India and Its States: Evidence From the National Family Health Survey 2015–16 and 2019–21

**DOI:** 10.7759/cureus.70733

**Published:** 2024-10-02

**Authors:** Damilola Ibirogba, Vishnu B Menon, Jeby Jose Olickal, Kavumpurathu R Thankappan

**Affiliations:** 1 Department of Public Health, Amrita Institute of Medical Sciences, Amrita Vishwa Vidyapeetham, Kochi, IND; 2 Department of Community Medicine, Amrita Institute of Medical Sciences, Amrita Vishwa Vidyapeetham, Kochi, IND

**Keywords:** adolescents, anemia, india, national family health survey, prevalence

## Abstract

Background: Limited research has been conducted on trends in anemia among a nationally representative sample of adolescents in India. We aimed to determine the trends in anemia prevalence and predictors of anemia among adolescents aged between 15 and 19 years in India and its different states.

Materials and methods: We utilized data from India's National Family Health Survey (NFHS-4) conducted during 2015-16 and NFHS-5 conducted during 2019-2021, comprising 237,446 adolescents aged between 15 and 19 years.

Results: The prevalence of anemia was 54% (95% CI 53.8-54.4) in NFHS-4 and 59.2% (CI 58.9-59.5) in NFHS-5. Twenty-one of 28 Indian states and five of eight union territories (UTs) reported an increase, depicting state-wise variation. While Assam, Jammu and Kashmir showed a substantial rise, the UTs of Lakshadweep, Andaman and Nicobar Islands recorded great declines. Younger age and rural residence were significant predictors of anemia (p<0.05) in NFHS-5 but not in NFHS-4.

Conclusion: Anemia prevalence among adolescents in India increased significantly in NFHS-5 compared to NFHS-4. Strategies to reduce anemia among adolescents need to focus on affected states and UTs, pregnant adolescents, those with unimproved sanitation, no education, and rural residents.

## Introduction

Anemia affects approximately 1.62 billion people worldwide, accounting for a quarter of the world's population (approximately 25%) [1, 2]. In 2019, the prevalence of anemia, adjusted for national age standards, varied between 3,118.0 and 49,327.1 cases per 100,000 population; countries like Zambia, Mali, and Burkina Faso reported the highest rates, while France, Iceland, and Belgium had the lowest rates [3]. The global prevalence of anemia among adolescents was 15% (27% in low- and middle-income countries (LMICS) and 6% in developed countries) [4]. India, having the most anemic adolescents in the world (72%), is followed by Jordan (69%), whereas Norway and the United States reported a much lower prevalence of around 5% [5].

The percentage change in age-standardized point prevalence showed a significant increase between 1990 and 2019 across various countries, with the highest rates observed among females aged 15-19 [6,7]. Notably, in western Sub-Saharan Africa, there were prevalent cases of dietary iron deficiency, with rates of 61.0% in males and 47.5% in females, while Andean Latin America exhibited even higher rates, with 74.1% in males and 58.1% in females [6,8]. On a global scale, the highest number of cases was primarily attributed to dietary iron deficiency (males: 66.1%, females: 56.8%), followed by hemoglobinopathies and hemolytic anemia (males: 13.6%, females: 16.1%) [9, 10].

Anemia has been associated with various physical complications, growth and cognitive impairment, reduced work capacity, increased susceptibility to infections, and high reproductive morbidity in adolescents and girls during their maturation into womanhood [11, 12]. For adolescent girls who embark on pregnancy (with approximately 21 million cases reported annually), anemia can have far-reaching implications, including maternal and neonatal mortality, as well as adverse birth outcomes [2, 13].

Anemia prevention entails increasing iron intake through diet, which can be difficult in developing countries [10]. Iron fortification of staple foods is being attempted in these areas to compensate for this deficiency [8]. Conversely, in developed countries, iron deficiency with or without anemia is almost always associated with diseases that cause a negative balance between iron absorption and loss [10, 11].

Projections indicate that by 2030, a total of 21 LMICs will continue to experience elevated levels of overall anemia. Furthermore, 61 nations are anticipated to have districts where anemia prevalence exceeds 40%, falling short of the World Health Organization's (WHO) Global Nutrition Target. In contrast, only three currently endemic countries (China, Iran, and Thailand) are predicted to successfully attain the Global Nutrition Target by the year 2030 [10].

To address this, WHO set a target of a 50% reduction in anemia in reproductive-age women by 2025; India had a national target to bring down the anemia prevalence to 40% in children (six to 59 months), 36% and 11% in adolescent (15-19 years) girls and boys, respectively, by 2022 [13]. The Anemia Mukt Bharat Program under the Prime Minister's Overarching Scheme for Holistic Nutrition, known as the POSHAN Abhiyaan, aims to reduce anemia prevalence by an annual 3% point among children aged between six and 59 months, adolescents, and women of reproductive age in India. [14]. Food fortification, intermittent iron supplementation, and parasitic infection control are key strategies that have underpinned the prevention and control of anemia and its related health challenges [15,16].

These strategies aside, the increased national burden of anemia across all demographics is a cause for concern within India's public health system [4,11,15].

The National Family Health Surveys (NFHS), conducted at five-year intervals, systematically capture data on anemia among adolescents. However, the literature on trends in anemia prevalence and predictors remains limited. Herein, we investigated the trends in anemia prevalence and predictors associated with adolescents across India and its diverse states and union territories (UTs).

## Materials and methods

Study design and population

This study is a secondary data analysis of two consecutive NFHSs conducted between 2015-16 and 2019-21. Both surveys (NFHS-4 and NFHS-5) adopted a two-stage stratified random sampling. For the selection of the rural samples, villages served as the primary sampling units (PSUs), followed by the random selection of 22 families in each PSU based on a probability proportional to their size. In contrast, Census enumeration blocks (CEB) were chosen at the first step of the two-stage sample design used in urban areas, before the random selection of 22 households from each CEB at the second stage [7]. During both NFHS-4 and NFHS-5 surveys, adolescents aged between 15 and 19, who willingly consented, provided finger-prick blood samples. These samples were analyzed using a battery-powered portable HemoCue Hb 201+ (HemoCue AB, Ängelholm, Sweden). The microcuvettes of the HemoCue 201+ were filled with drops of capillary blood for hemoglobin estimation. Findings were then documented in the Biomarker Questionnaire [13].

Statistical analysis

In this study, the dependent variable was the presence or absence of anemia among the respondents. Weights in the sample are calculated by multiplying the household weight by the inverse of the individual response rates in the stratum [13]. Among girls who were not pregnant, anemia was identified by hemoglobin levels falling between 11.0 and 11.9 g/dL, while for pregnant girls, it was between 10.0 and 10.9 g/dL [11]. The study created a binary variable to indicate the prevalence of anemia. Adolescents with anemia were assigned a code of '1', while those without were assigned a code of '0'.

The sociodemographic profile of the adolescent sample was then accounted for across various covariates. This was followed by a comprehensive assessment of prevalence separately for the survey periods of 2015-16 and 2019-21. Given that national-level analyses mask regional disparities, we further explored the anemia prevalence for individual states in both periods.

To investigate the independent effects of factors associated with anemia, we employed a multivariable binary logistic regression analysis. A pooled estimate of data from both NFHS-4 and NFHS-5 not only increased the statistical power and sample size but also confirmed the independent impact of the survey year on the likelihood of anemia.

Before conducting the multivariable binary logistic regression model, a chi-square analysis was performed to assess the association between each independent variable and anemia. Variables that displayed a statistically significant bivariate relationship with the outcome were subsequently incorporated into the ensuing multivariable logistic regression analysis.

Given that our study included multiple explanatory variables that could potentially be correlated, we computed variance influence factors (VIF) to evaluate the presence of multicollinearity among the independent variables (all VIFs < 5) [13]. To estimate the goodness of fit, we examined the pseudo-R-squared value and the Hosmer-Lemeshow goodness-of-fit test and also considered the log-likelihood. This resulted in a non-significant result, suggesting a good fit between the model and the data [13].

Given the complex sampling design and incorporation of sample weights in the analyzed surveys, we applied complex sample analysis in IBM SPSS software for Windows, version 28.0 (IBM Corp., Armonk, NY), creating specific plan files for each survey. Adjusted odds ratios (AOR) along with their corresponding 95% confidence intervals (CI) were computed, and a p-value below 0.05 was deemed statistically significant. The analyses were conducted using IBM SPSS Statistics for Windows, version 28.0. For visual representations, choropleth maps were created using Datawrapper 2023 (Datawrapper GmbH, Berlin, Germany).

Ethical considerations

Ethical approval for this study was provided by the ethical committee of Amrita School of Medicine, Kochi, India (approval number: ECASM-AIMS-2023-111) on 24-02-2023.

## Results

Profiles of the respondents

Table 1 provides an overview of the sociodemographic profile of adolescents aged between 15 and 19 years in both NFHS-4 and NFHS-5, with a combined sample size of N = 237,446. A higher proportion of adolescents (59.5%) were in the 15-17 age group, belonged to the category of Other Backward Classes (46.3%), possessed educational qualifications (93.5%), never had been married (84.5%), resided in rural areas (70%), and adhered to the Hindu faith (79.08%). Notably, we observed an upward trend in these proportions as we moved from NFHS-4 to NFHS-5.

**Table 1:** Sociodemographic characteristics of adolescents aged between 15 and 19 years (N = 237,446) NFHS: National Family Health Survey

Variables	NFHS-4 (121,730)	NFHS-5 (115,716)
	n (%)	n (%)
Age (years)		
15-17	73,158 (59.5)	69,116 (59.56)
18-19	48,572 (40.5)	46,600 (40.44)
Caste		
Other Backward Class (OBC)	49,400 (46.3)	45,368 (46.04)
Scheduled Caste	23,081 (22.4)	23,768 (24.36)
Others	22,735 (21.6)	19,316 (19.42)
Scheduled Tribe	21,710 (9.9)	21,724 (10.19)
Educational level		
Educated	113,179 (93.2)	110,701 (95.73)
No education	8,551 (6.8)	5,015 (4.27)
Marital status		
Never married	104,986 (84.5)	102,510 (87.01)
Married	16,744 (15.5)	13,206 (12.99)
Wealth Index		
Poorest	26,040 (20.92)	25,086 (21.71)
Poorer	29,507 (22.75)	26,662 (22.62)
Middle	26,702 (21.51)	24,542 (21.12)
Richer	22,161 (19.08)	21,566 (19.10)
Richest	17,320 (15.73)	17,860 (14.45)
Type of residence		
Rural	89,748 (70.24)	90,904 (72.69)
Urban	31,982 (29.76)	24,812 (27.31)
Religion		
Hindu	89,496 (79.08)	87,049 (80.37)
Muslim	19,122 (16.16)	16,083 (15.42)
Christian	8,250 (1.94)	7,798 (2.01)
Others	4,786 (2.82)	4913 (2.21)
Pregnancy		
Not pregnant	118,123 (96.73)	112,837 (97.24)
Pregnant	3,607 (3.27)	2,879 (2.76)

**Figure 1:**
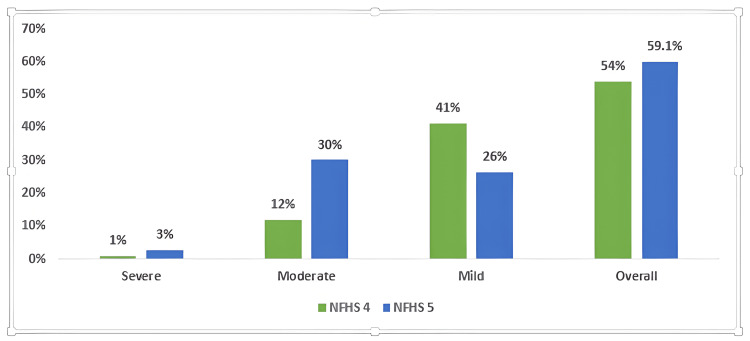
Prevalence (%) of anemia based on severity in NFHS-4 and NFHS-5 NFHS: National Family Health Survey

The overall estimate of anemia prevalence in adolescents as depicted in Figure 1 increased by 5.1% (54% in NFHS-4 to 59.1% in NFHS-5). In terms of severity, a 50% decline in mild anemia, which was offset by one in every three adolescents being moderately anemic, was observed. Approximately 3% of adolescents were categorized as severely anemic.

Interstate variability and percentage change in anemia prevalence among adolescents

**Figure 2:**
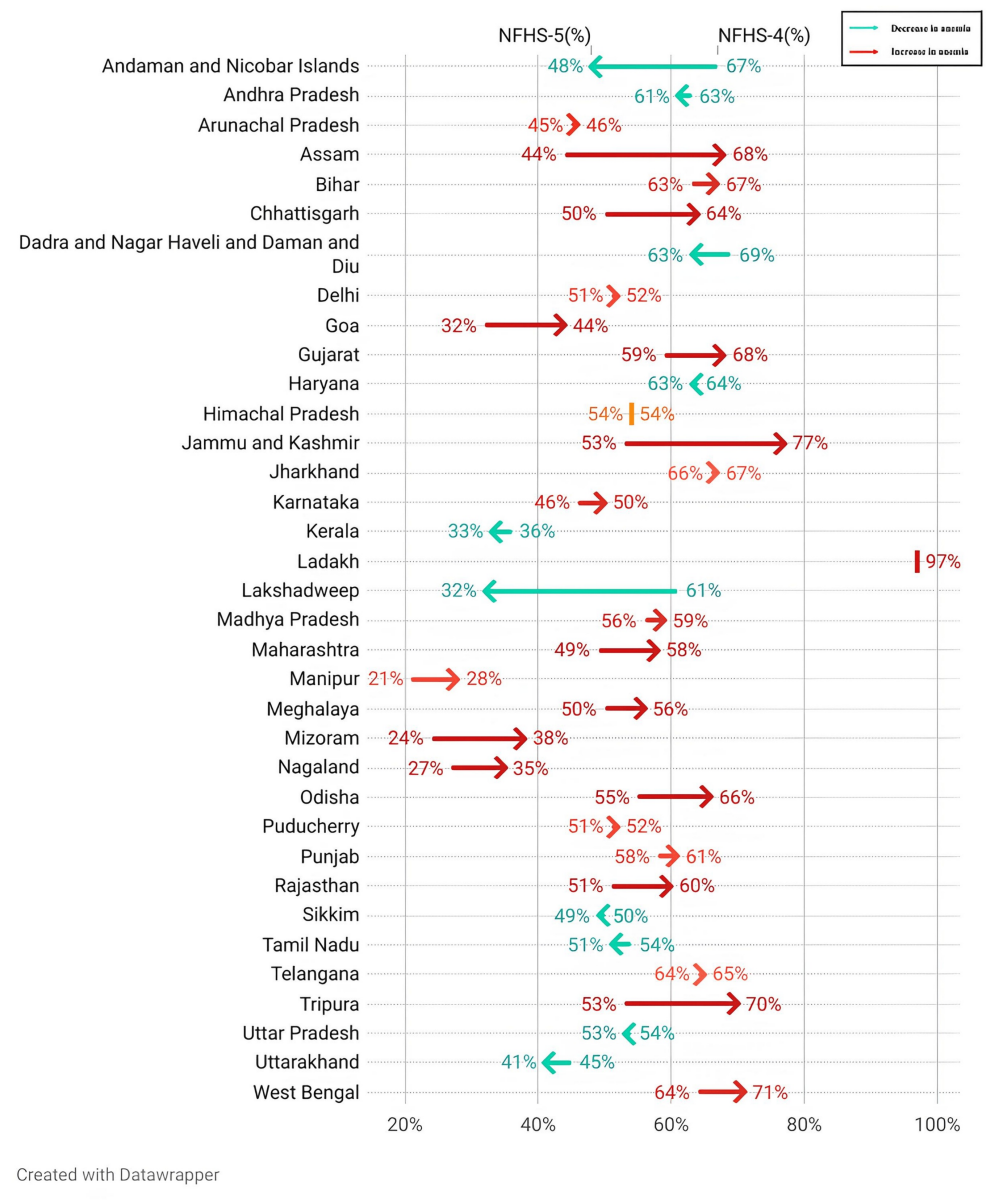
Interstate variability in anemia prevalence among adolescents and percentage change (NFHS-4 and NFHS-5) NFHS: National Family Health Survey This image was created by the first author using Datawrapper (Datawrapper GmbH, Berlin, Germany)

Figure 2 below provides a subnational analysis of prevalence, which sees a reduction in only seven major states and an increase in 21 others. Of the eight UTs, anemia prevalence declined in only three. This provides empirical evidence regarding the progress and setbacks made by various states in combating anemia during the surveyed periods.

Estimates from logistic regression analysis for anemia among adolescents in India

Bivariate analyses revealed that type of residence, caste, level of education, marital status, wealth index, type of water source, sanitation type, media exposure, blood sugar, and pressure were statistically associated with anemia. The results are presented in Table 2.

**Table 2:** Factors associated with the prevalence of anemia among adolescents aged between 15 and 19 years in NFHS-4 and NFHS-5: results of bivariate analysis NFHS: National Family Health Survey

Variables	NFHS-4 anemic n (%)	p-value	NFHS-5 anemic n (%)	p-value
Age (years)				
15-17	38,300 (54.4)	0.753	40,790 (59.82)	<0.001*
18-19	25,384 (54.6)		27,015 (58.15)	
Residence type				
Rural	47,715 (54.67)	<0.001*	54,297 (60.15)	<0.001*
Urban	15,969 (52.67)		13,508 (56.47)	
Caste				
Scheduled Caste	12,844 (56.44)		14,416 (61.04)	
Scheduled Tribe	11,410 (60.06)	<0.001*	13,084 (67.04)	<0.001*
Other Backward Class (OBC)	25,985 (52.96)		25,809 (56.65)	
Others	11,084 (52.13)		10,988 (57.55)	
Level of education				
Educated	58,594 (59.63)	<0.001*	64,537 (58.89)	<0.001*
No education	5,090 (53.67)		3,268 (64.78)	
Marital status				
Married	9,411 (56.59)	<0.001*	8,151(63.16)	<0.001*
Never married	54,273 (53.61)		59,654 (58.54)	
Religion				
Christian	2,970 (47.46)		3,635 (53.26)	
Hindu	48,324 (54.69)	<0.001*	51,662 (59.38)	<0.001*
Muslim	9,809 (51.82)		9,716 (58.33)	
Others	2,581 (54.36)		2,792 (61.38)	
Wealth index				
Poorest	15,087 (58.31)		15,736 (63.95)	
Poorer	15,425 (54.91)		15,837 (60.54)	
Middle	13,613 (53.80)	<0.001*	14,201 (58.74)	<0.001*
Richer	11,195 (52.12)		12,253 (56.65)	
Richest	8,364 (49.97)		9,778 (53.96)	
Type of water source				
Improved water source	55,244 (54.08)	0.106	59,568 (59.08)	0.178
Unimproved water source	8,440 (54.05)		8,237 (59.64)	
Sanitation				
Improved sanitation	30,717 (52.12)	<0.001*	49,106 (57.92)	<0.001*
Unimproved sanitation	32,967 (56.13)		18,699 (62.50)	
Media exposure (radio, TV, newspaper)				
No	16,599 (57.32)	<0.001*	18,538 (62.64)	<0.001*
Yes	47,085 (53.07)		49,267 (57.93)	
Blood sugar				
High	1,465 (59.99)		1,669 (65.46)	<0.001*
Low	1,327 (52.07)	<0.001*	310 (59.43)	
Normal	60,879 (54.00)		65,811 (58.97)	
Body mass index (BMI)				
Normal	37,313 (52.2)		39,969 (58.6)	
Obese	2,243 (44.5)	<0.001*	3,135 (51.5%)	<0.001*
Underweight	26,720 (55.3)		26,743 (61.2%)	
Blood pressure				
Elevated	763 (47.46)	<0.001*	948 (52.52)	
Normal	62,842 (54.17)		66,730 (59.25)	
Pregnancy status				
Not pregnant	61,836 (54.13)	0.187	66,222 (59.19)	<0.001*
Pregnant	1,848 (52.41)		1,583 (57.34%)	

All significant independent variables associated with anemia prevalence were further analyzed using multivariable binary logistic regression. The results are presented in Tables 3-4.

**Table 3:** Predictors of anemia among adolescents aged between 15 and19 years in NFHS-4: results of multivariable logistic regression analysis NFHS: National Family Health Survey

	NFHS-4			
Variables	Adjusted odds ratio	95% CI		p-value
		Lower	Upper	
Resident type				
Rural	1.03	1.001	1.06	.0818
Urban	1			
Caste				
Scheduled Caste	1.24	1.19	1.29	<0.001
Scheduled Tribe	1.36	1.49	1.086	<0.001
Other Backward Class (OBC)	1.11	1.08	1.15	<0.001
Others	1			
Educational level				
No education	1.17	1.12	1.23	<0.001
Educated	1			
Marital status				
Married	1.12	1.08	1.16	<0.001
Never married	1			
Sanitation status				
Unimproved	1.11	1.07	1.14	<0.001
Improved	1			
Blood pressure				
Normal	1.39	1.26	1.54	<0.001
Elevated	1			
Religion				
Hindu	2.35	2.22	2.48	<0.001
Muslim	2.29	2.15	2.45	<0.001
Others	2.23	2.07	2.40	<0.001
Christian	1			
Wealth index				
Poorest	1.13	1.07	1.20	<0.001
Poorer	1.01	0.96	1.06	0.797
Middle	1.02	0.97	1.06	0.399
Richer	1.05	1.01	1.10	0.016
Richest	1			
Blood sugar				
Normal	1.05	0.97	1.14	0.23
Elevated	1.33	1.19	1.49	<0.001
Low	1			
Media exposure (radio, TV, newspaper)				
No	1.03	0.997	1.07	0.072
Yes	1			

**Table 4:** Predictors of anemia among adolescents aged between 15 and 19 years in NFHS-5: results of multivariable logistic regression analysis NFHS: National Family Health Survey

	NFHS-5			
Variables	Adjusted odds ratio	95% CI		p-value
		Lower	Upper	
Age (in years)				
15-17	1.06	1.04	1.10	<0.001
18-19	1			
Resident type				
Rural	1.20	1.16	1.23	<0.001
Urban	1			
Caste				
Scheduled Caste	1.17	1.13	1.21	<0.001
Scheduled Tribe	1.35	1.30	1.40	<0.001
Others	1.05	1.01	1.09	0.003
Other Backward Class (OBC)	1			
Educational level				
No education	1.14	1.07	1.21	<0.001
Educated	1			
Marital status				
Married	1.21	1.16	1.27	<0.001
Never married	1			
Sanitation status				
Unimproved	1.08	1.05	1.12	<0.001
Improved	1			
Blood pressure				
Normal	1.29	1.17	1.42	<0.001
Elevated	1			
Religion				
Hindu	1.98	1.88	2.09	<0.001
Muslim	2.17	2.03	2.32	<0.001
Others	1.77	1.64	1.91	<0.001
Christian	1			
Wealth index				
Poorest	1.27	1.21	1.33	<0.001
Poorer	1.15	1.11	1.20	<0.001
Middle	1.10	1.05	1.14	<0.001
Richer	1.07	1.03	1.12	0.001
Richest	1			
Blood sugar				
Low	1.15	0.95	1.37	0.136
Elevated	1.31	1.20	1.42	<0.001
Normal	1			
Media exposure (radio, TV, newspaper)				
No	1.01	0 .974	1.04	0.698
Yes	1			
Pregnant				
No	1.41	1.29	1.53	<0.001
Yes	1			

Adolescents belonging to the Scheduled Caste, Scheduled Tribe, and Other Backward Classes had a higher likelihood of being anemic. A similar pattern, although slightly higher, persisted in NFHS-5. In both NFHS-4 and NFHS-5, uneducated adolescents were about 1.2 times more predisposed to anemia as compared to educated adolescents (adjusted odds ratio (aOR) 1.17, CI 1.12-1.23, p-value < 0.001). Adolescents living in conditions of unimproved sanitation are 1.1 times more likely to be anemic (aOR 1.11, CI 1.07-.1.14, p-value < 0.001). With a lesser odd, a similar trend was observed in NFHS-5. Anemia susceptibility varied significantly with religion in NFHS-4, as evidenced by aOR > 2 (95% CI 2.22-2.48, p-value < 0.001). Nevertheless, these odds reduced in NFHS-5.

## Discussion

One major finding of this study was that the overall prevalence of anemia among Indian adolescents (aged between 15 and 19 years) significantly increased from 54% in 2015-16 to 59.1% in 2019-21. This compares to NFHS results released by the Ministry of Health and Family Welfare (MoHFW) but is higher than those reported by Sarna et al. [16]. Between 2000 and 2019, however, there was a general downward trend in prevalence in the context of worldwide distribution [17, 18]. Here, high-income countries experienced an increase while there was a decrease in their low-income counterparts [18].

Of the 28 major Indian states (each with a population exceeding 10 million), the prevalence of anemia reduced in only seven. Among the nation’s eight UTs, prevalence declined in three. Studies conducted on women and adolescent girls exist to give credence to this subnational disparity in prevalence [4, 19-20].

To combat anemia, Kerala implemented state-specific strategies, including school-based iron supplements, opportunistic hemoglobin screening, promoting iron-rich diets and exercise, introducing iron-rich kitchen gardens, and conducting door-to-door campaigns [19, 21]. While the Ladakh administration is committed to supplying its residents with fortified wheat, Dadra & Nagar Haveli and Daman & Diu have embraced an integrated strategy like the Swabhiman initiative to effectively address this issue [22, 23]. The archipelago of Lakshadweep followed the Anemia Mukt Bharat (AMB) program, incorporating prophylactic iron and folic acid supplementation along with deworming [22]. The AMB program implementation in states like Assam was impacted by the COVID-19 pandemic [22].

In 2015-16, significant factors linked with anemia prevalence were caste, educational attainment, marital status, wealth index, sanitation, media exposure, blood sugar levels, and blood pressure levels. However, in the 2019-21 period, age, pregnancy status, and residence type were added to the list of statistically significant contributors, indicating a persistent relationship between existing determinants and anemia prevalence across both timeframes. Water source type was an insignificant determinant in both surveys.

Anemia prevalence increased with increasing age in both reporting periods [23]. It should, however, be noted here that this increase was significant in the fifth round of the survey but not in NFHS-4.

Anemia in adolescents exhibited caste-wise disparity. Scheduled caste, scheduled tribe, and other backward caste adolescents have a higher likelihood of being anemic. A similar pattern, although slightly lesser, persisted in NFHS-5. This calls for the need for sustained targeted interventions and policies to address these caste-related disparities in anemia and promote equitable health outcomes among different caste groups [24].

Adolescents' susceptibility to anemia seems to differ depending on the religious groups they are affiliated with. When compared to Christians, adolescents of other religions are approximately two times more likely to be anemic. It then becomes essential to approach this on a case-by-case basis and consider various factors such as dietary habits, access to healthcare, and individual beliefs within specific religious contexts [25].

Pregnancy was interlinked with anemia as a predictor in NFHS-5 but not in NFHS-4. This further emphasizes the need for targeted interventions and comprehensive healthcare support to address this significant health concern [26].

Education is a protective factor against anemia. In both NFHS-4 and NFHS-5, adolescents with no education were more likely to be anemic as compared to educated adolescents. Previous studies from Ethiopia and India have found a strong association between education and anemia [27]. Although this demographic represents a developing cohort, the impact of education on their overall health outcome cannot be overemphasized.

Unimproved sanitation predisposes to anemia. In NFHS-4, inadequate sanitation conditions were associated with a 1.11 times higher likelihood of anemia among adolescents. Interestingly, this risk appears to be somewhat reduced in NFHS-5. The underlying biological mechanism here involves elevated hepcidin levels, which, in turn, inhibit the absorption of iron in the intestines and contribute to iron deficiency [28-30]. This emphasizes the positive impact of improved sanitation practices on anemia and the promotion of a co-benefit of sanitation programs.

Finally, this study showed that we have more anemic adolescents in rural India compared to urban India and a further increasing trend in NFHS-5, with the difference being statistically significant. This finding contrasts with results from the Comprehensive National Nutrition Survey 2016-18 that reported a higher prevalence in urban than in rural participants [30]. The observed rural-urban disparity in anemia may be attributed to reverse migration prevalent during the COVID-19 lockdown [30]. As these adolescents returned to their rural homes due to the lockdown, they might have encountered challenges related to healthcare access, nutrition, and economic stability, which could have contributed to an increase in anemia cases in these areas [30].

Limitations

National Family Health Surveys rely on capillary blood samples to assess hemoglobin levels. This approach carries the possibility of resulting in elevated hemoglobin levels. Also, adolescents within the age range of 10-14 years were not captured in NFHS. In addition, because the study is cross-sectional, it is not possible to determine a causal relationship between the independent variables and anemia. Finally, we do not have information about several diet-related determinants, such as folate, vitamin B12, and vitamin A, in the dataset. These may also influence the prevalence of anemia.

Recommendations

Despite advancements in science and medicine, anemia remains a pressing issue due to political, socioeconomic, and behavioral factors in India. To address this, a nationwide survey using venous blood samples should be a top priority for the Indian government to assess anemia prevalence and its determinants in adolescents aged between 10 and 19 years. Additionally, interventions should be targeted at discouraging indoor smoking, and diagnostic cutoffs should be reviewed. Finally, emphasizing mobile Health (mHealth) for tech-savvy adolescents and implementing peer educator change agents could further bolster the fight against anemia.

## Conclusions

With an overall increase in anemia prevalence among adolescents, the findings of this study underpin anemia as a pressing national public health issue among adolescents. A subnational analysis showed a reduction in only seven major states, while 21 states experienced an increase. Among the eight UTs, anemia prevalence declined in just three. Adolescents who are uneducated, lack access to improved sanitation, or belong to the scheduled caste, scheduled tribe, or other backward castes are at a higher risk of being anemic. This necessitates a reevaluation and adaptation of existing anemia programs to prioritize state-wise disparities and highlight vulnerable groups.

## References

[1] Aspuru K, Villa C, Bermejo F, Herrero P, López SG (2011). Optimal management of iron deficiency anemia due to poor dietary intake. Int J Gen Med.

[2] Verma K, Baniya GC (2022). Prevalence, knowledge, and related factor of anemia among school-going adolescent girls in a remote area of western Rajasthan. J Family Med Prim Care.

[3] Kassebaum NJ (2016). The global burden of anemia. Hematol Oncol Clin North Am.

[4] Chandrakumari AS, Sinha P, Singaravelu S, Jaikumar S (2019). Prevalence of anemia among adolescent girls in a rural area of Tamil Nadu, India. J Family Med Prim Care.

[5] Prasanth R (20172023). Prevalence of anemia in both developing and developed countries around the world. World J Anemia.

[6] Safiri S, Kolahi AA, Noori M (2021). Burden of anemia and its underlying causes in 204 countries and territories, 1990-2019: results from the Global Burden of Disease Study 2019. J Hematol Oncol.

[7] Neena Bhatia (2022). Analysis of key nutrition indicators based on National Family Health Survey, NFHS 4 (2015-16) and NFHS 5 (2019-2021). https://www.academia.edu/84405957/Analysis_of_Key_Nutrition_Indicators_Based_on_National_Family_Health_Survey_NFHS_4_2015_16_and_NFHS_5_2019_2021_?uc-sb-sw=70367316.

[8] Tesfaye M, Yemane T, Adisu W, Asres Y, Gedefaw L (2015). Anemia and iron deficiency among school adolescents: burden, severity, and determinant factors in southwest Ethiopia. Adolesc Health Med Ther.

[9] Kinyoki D, Osgood-Zimmerman AE, Bhattacharjee NV, Kassebaum NJ, Hay SI (2021). Anemia prevalence in women of reproductive age in low- and middle-income countries between 2000 and 2018. Nat Med.

[10] Wiafe MA, Ayenu J, Eli-Cophie D (2023). A review of the risk factors for iron deficiency anaemia among adolescents in developing countries. Anemia.

[11] Scott S, Lahiri A, Sethi V (2022). Anaemia in Indians aged 10-19 years: prevalence, burden and associated factors at national and regional levels. Matern Child Nutr.

[12] Merid MW, Chilot D, Alem AZ, Aragaw FM, Asratie MH, Belay DG, Kibret AA (2023). An unacceptably high burden of anaemia and it's predictors among young women (15-24 years) in low and middle income countries; set back to SDG progress. BMC Public Health.

[13] Let S, Tiwari S, Singh A, Chakrabarty M (2023). Prevalence and determinants of anaemia among women of reproductive age in aspirational districts of India: an analysis of NFHS 4 and NFHS 5 data. BMC Public Health.

[14] Belwal E, Pandey S, Sarkar S (2021). Anemia prevalence in India over two decades: evidence from National Family Health Survey (NFHS). Int J Sci Healthcare Res.

[15] Rai RK, Kumar SS, Sen Gupta S (2023). Shooting shadows: India's struggle to reduce the burden of anaemia. Br J Nutr.

[16] Sarna A, Porwal A, Ramesh S (2020). Characterisation of the types of anaemia prevalent among children and adolescents aged 1-19 years in India: a population-based study. Lancet Child Adolesc Health.

[17] Stevens GA, Paciorek CJ, Flores-Urrutia MC (2022). National, regional, and global estimates of anaemia by severity in women and children for 2000-19: a pooled analysis of population-representative data. Lancet Glob Health.

[18] Roy N, Mishra VK, Mishra PK, Bandhu A, Dhakad R (2021). Changing scenario of anemia among women in India: understanding the maternal health concern and its associated predictors through National Family Health Survey. Res Square.

[19] Maya C (2023). Kerala plans steps to tackle iron-deficiency anemia in women. https://www.thehindu.com/news/national/kerala/kerala-plans-steps-to-tackle-iron-deficiency-anaemia-in-women/article66104593.ece.

[20] Patel SP (2023). Integrated approach for malnutrition & anaemia-free Dadra and Nagar Haveli & Daman and Diu. Hon’ble Prime Minister of India. [Internet]. [Cited.

[21] (2023). Come April, Ladakh administration to provide residents with fortified wheat to fight anaemia. https://www.hindustantimes.com/cities/chandigarh-news/come-april-ladakh-administration-to-provide-residents-with-fortified-wheat-to-fight-anaemia-101673509912040.html.

[22] (2023). Anaemia Mukt Bharat. https://pib.gov.in/PressReleaseIframePage.aspx?PRID=2042053.

[23] Cappellini MD, Motta I (2015). Anemia in clinical practice-definition and classification: does hemoglobin change with aging?. Semin Hematol.

[24] Vart P, Jaglan A, Shafique K (2015). Caste-based social inequalities and childhood anemia in India: results from the National Family Health Survey (NFHS) 2005-2006. BMC Public Health.

[25] Sagalova V, Vollmer S, Ntambi J (2021). Socio-economic predictors of undernutrition and anaemia in adolescent mothers in West and Central Africa. J Glob Health.

[26] Sunuwar DR, Singh DR, Chaudhary NK, Pradhan PM, Rai P, Tiwari K (2020). Prevalence and factors associated with anemia among women of reproductive age in seven South and Southeast Asian countries: evidence from nationally representative surveys. PLoS One.

[27] Muchomba FM (2022). Effect of schooling on anemia and nutritional status among women: a natural experiment in Ethiopia. Am J Epidemiol.

[28] Baldi AJ, Clucas D, Pasricha SR (20202023). Anemia and water, sanitation, and hygiene (WASH)—is there really a link?. Am J Clin Nutr [Internet.

[29] Kothari MT, Coile A, Huestis A, Pullum T, Garrett D, Engmann C (2019). Exploring associations between water, sanitation, and anemia through 47 nationally representative demographic and health surveys. Ann N Y Acad Sci.

[30] Kulkarni B, Peter R, Ghosh S (2021). Prevalence of iron deficiency and its sociodemographic patterning in Indian children and adolescents: findings from the Comprehensive National Nutrition Survey 2016-18. J Nutr.

